# Spatio-temporal characteristics of the novel coronavirus attention network and its influencing factors in China

**DOI:** 10.1371/journal.pone.0257291

**Published:** 2021-09-16

**Authors:** Xiaojia Guo, Jing Zhang, Xueling Wu

**Affiliations:** 1 College of Geographical Science, Shanxi Normal University, Taiyuan, Shanxi, China; 2 Institute of Geosciences and Resources, Chinese Academy of Sciences, Beijing, China; Northeastern University (Shenyang China), CHINA

## Abstract

The outbreak of a novel coronavirus pneumonia (COVID-19), wherein more than 200 million people have been infected and millions have died, poses a great threat to achieving the United Nations 2030 sustainable development goal (SDGs). Based on the Baidu index of ’novel coronavirus’, this paper analyses the spatial and temporal characteristics of and factors that influenced the attention network for COVID-19 from January 9, 2020, to April 15, 2020. The study found that (1) Temporally, the attention in the new coronavirus network showed an upward trend from January 9 to January 29, with the largest increase from January 23 to January 29 and a peak on January 29, and then a slow downward trend. The level of attention in the new coronavirus network was basically flat when comparing January 22 and March 4. (2) Spatially, first, from the perspective of regional differences, the network attention in the eastern and central regions decreased in turn. The network users in the eastern region exhibited the highest attention to the new coronavirus, especially in Guangdong, Shandong, Jiangsu and other provinces and cities. The network attention in Tibet, Xinjiang, Qinghai and Ningxia in the western region was the lowest in terms of the national network attention. Second, from the perspective of interprovincial differences, the attention in the new coronavirus network was highly consistent with the Hu Huanyong line of China’s population boundary. The east of the Hu Huanyong line is densely populated, and the network showed high concern, mostly ranking at the third to fifth levels. (3) The number of Internet users in the information technology field, the population, and the culture and age characteristics of individuals are important factors that influence the novel coronavirus attention network.

## 1. Introduction

On February 11, 2020, the World Health Organization (WHO) named the disease caused by the novel coronavirus COVID-19 [1], which had a comprehensive impact on global social and economic life [2]. To date, more than 200 million people have been infected worldwide, resulting in millions of deaths. Around the world, the top five epidemic severity indexes were observed in the UK, France, Sweden, the Netherlands and Belgium. Throughout human history, society has been challenged with infectious diseases, natural disasters and other emergencies [3]. China, in particular, is in a critical period of social transformation and modernization. However, inevitable trends in global urbanization, rapid urbanization and rural revitalization have remodelled the human environment and caused radical climate changes [4, 5]. In particular, the implementation of high-speed rail transport in China has accelerated personnel flow and caused unpredictable problems [6], increasing the difficulty of COVID-19 prevention. It is well known that the occurrence of communicable diseases depends on three key factors: the infection source, the transmission route and the susceptible population [7]. However, due to the high concentration of urban populations, global factors, frequent regional linkages and the lack of effective emergency response mechanisms, the transmission route has become the most difficult and uncontrollable. There is no doubt that the high frequency of major public health events is creating new challenges for the national governance system [8]. President Xi Jinping noted that the new coronavirus pneumonia epidemic was "a major public health emergency with the fastest spread, the widest range of infection and the most difficult prevention and control since the founding of the People’s Republic of China".

When the epidemic was raging, it was essential to perform orderly, coordinated, timely and comprehensive research. At present, foreign research on the novel coronavirus epidemic has focused mostly on the clinical characteristics of patients with COVID-19 [9–11], chest characteristics shown by computed tomography (CT) [12–14], the treatment of severe patients [15–17], the influence of COVID-19 on other diseases [18, 19], the monitoring of the novel coronavirus epidemic [20, 21], and novel coronavirus vaccines [22–25]. According to the history of epidemiology, careful investigations of exposure environments and exposed populations are necessary to isolate potential sources of transmission and prevent second-generation transmission. With the popularity of the Internet and the development of geographic information technology, network information flows have provided various ways for people to learn and obtain information, and networks have become an important channel for gaining knowledge [26].

In the prevention and control of the novel coronavirus pneumonia epidemic in China, the release of epidemic information across the country has been timely, accurate, open and transparent. People can immediately obtain real-time dynamic information related to the epidemic and action paths for infected individuals. Additionally, applications (apps) such as WeChat and Alipay can be used to effectively reduce panic levels and the spread of rumours related to the epidemic. The degree of attention from Internet users toward an event or thing on the Internet is usually expressed by the frequency of Internet users’ search indexes. The higher the search frequency is, the higher the degree of attention of Internet users to an event, and vice versa.

Search indexes can be obtained from different platforms, such as the Baidu Index, Google Trends, Sina Weibo [27], short video indexes, and social network indexes [28]. Chinese scholars Tian and Li used spatial correlation and spatial regression analysis methods to explore the temporal and spatial evolution characteristics of and factors that influence the Danxia Mountain attention network based on Baidu index data [29]. Shi and Lan used the average daily tourism search indexes of Shandong coastal cities to study the Shandong coastal tourism attention network. The study found that the seasonal variations in the Shandong coastal tourism attention network are obvious. The spatial distribution of network attention is relatively concentrated. Climate, the economy, the population, distance, information and other factors have an important influence on the attention networks of tourism destinations [30]. Li and Zhao et al. analysed the hot spots of "novel coronavirus pneumonia in traditional Chinese medicine" during the outbreak of the novel coronavirus through information from microblogs mined with an LDA model to understand the scientific connotation of traditional Chinese medicine in the public network, expose the potential threat of the development of traditional Chinese medicine, and prevent alienation and deviation [31].

Foreign scholar Bernstein et al. found Altmetric Attention Scores (AAS) to be an indicator of online attention and influence as well as Twitter, Facebook and news media references. Multivariate linear regression analysis was conducted to determine which manuscript factors were related to AAS. Then, multivariate linear regression analysis was performed to determine which manuscript factors were associated with AAS [32]. Badran and Hassona tried to identify research articles related to cleft lip and/or cleft palate (CL/P) that generated the highest online attention. Altmetric Explorer was used to identify the 100 articles with the highest Altmetric Attention Score (AAS) and was used to study the characteristics of these articles in relation to their publication data, research type and domain, number of Mendeley readers, and dimension citations [33]. Prange Philipp found that online investor attention measured by Google search could provide valuable information to assess linkages between financial assets [34].

In this study, the novel coronavirus was used as the keyword, and the time span was from January 9, 2020 (when the pathogen was preliminarily determined to be COVID-19), to April 14, 2020 (when the ICU ward of Wuhan Jinyintan Hospital announced that they had ’zero COVID-19 patients’; a monumental victory in the control of COVID-19) [35, 36]. Based on the Baidu index, the spatial and temporal differences of and factors that influence the novel coronavirus attention network were analysed, and the spreading trend and severity of the epidemic in various provinces in China were determined to scientifically and accurately assess the form of the national epidemic and provide a basis for promoting the precise prevention and control of the epidemic.

The research content of this paper is compared with some domestic studies: the research time is concentrated in the early outbreak of the epidemic to the basic stability of the epidemic within three months. The study of this paper shortens the research time. Through the analysis of the attention in the new coronavirus network, it can be concluded that in the most serious period of epidemic development, the change in people’s attention to the epidemic in different regions represents the attention of people at different stages of the epidemic and provides targeted, accurate and specific theoretical support for the normalization of epidemic prevention and control and other major public health emergencies in the future.

## 2. Methodology and data source

### 2.1 Research methods

Based on the attention networks in 31 provincial-level administrative regions (excluding Hong Kong, Macao and Taiwan) in China, an attention network for the public in China was developed using the keyword ’novel coronavirus’ by incorporating information from the Baidu index website from January 9, 2020, to April 15, 2020. Some general domestic research methods, such as the annual variation index, seasonal variation index, and weekly distribution skewness index, are limited by the time period of this study. The coefficient of variation (CV), geographic concentration index (Ge) and Gini coefficient (Gi) were used to analyse the temporal and spatial differences in the novel coronavirus attention network.

1. The geographical concentration index is used mainly to analyse the spatial distribution characteristics of a studied variable, and its formula is:
  $$\mathrm{Ge}=100\times \sqrt{\sum_{i=1}^{n}{\left(\frac{x_{i}}{T}\right)}^{2}}$$ (1)
  In Eq (1), *x_i_* is the search index for the novel coronavirus in the ith province, and T is the sum of the National Novel Coronavirus Baidu Search Index, where n = 31. The larger the value of Ge is, the more concentrated the novel coronavirus attention network, and the smaller the value of Ge is, the more dispersed the novel coronavirus attention network.
2. The Gini coefficient is used in quantitative geography to analyse the spatial distribution differences among variables within a discrete region and is represented by Gi. This index is expressed as follows:
  $$Gi=(-\sum_{i=1}^{n}X_{i}\times \ln X_{i})/lnN$$ (2)
  In formula (2), *x_i_* refers to the proportion of the attention network corresponding to the ith province relative to the overall attention network, N refers to the 31 provinces studied in this paper, and the calculated Gi value range is 0–1. If the Gi value is large, the spatial distribution of the novel coronavirus attention network is unbalanced, and the higher the concentration degree is, the smaller the Gi value and the more dispersed the distribution [37].
3. The coefficient of variation CV is a relative statistic used to measure the discreteness of different samples. This variable is the ratio of the standard deviation of a set of data to the corresponding average value. In this paper, the coefficient of variation is used to measure the degree of discreteness of temporal variations in the attention degree of the novel coronavirus network. The corresponding formula is as follows.
  $$\mathrm{CV}=\frac{\sqrt{\sum_{i=1}^{n}{\left(y_{i}-\bar{y}\right)}^{2}/n}}{\bar{y}}$$ (3)
  In formula (3), CV is the coefficient of variation, *y*_*i*_ is the attention in time period i, and $\bar{y}$ is the average value of *y*_*i*_. The greater the CV value is, the greater the time differences in the novel coronavirus attention network.
4. Moran’s I index, which reflects the similarity of attribute values in regional units that are adjacent in space, is commonly used to measure global and local spatial autocorrelations. The former reflects the overall agglomeration degree of observation indexes in a certain space, and the latter reflects the agglomeration degree of observation indexes between two units within a given space.
  $$I=\frac{n\sum_{i=1}^{n}\sum_{j=1}^{n}w_{ij}(x_{i-}-\bar{X})(x_{j}-\bar{X})}{\sum_{i-1}^{n}\sum_{j=1}^{n}w_{ij}\sum_{i=1}^{n}{\left(x_{i}-\bar{x}\right)}^{2}}$$
  $$I_{i}=\frac{\left(x_{i}-\bar{x}\right)}{S^{2}}\sum_{j}w_{ij}(x_{j}-\bar{x});$$ (4)
  In the formula, *I* represents the global spatial autocorrelation index and *I_i_* represents the local spatial autocorrelation Moran’s I index. *i* and *j* are provinces (plus districts and cities). This paper uses GeoDa software to calculate Moran’s I index.
5. Multivariate stepwise linear regression analysis is based on the principle of introducing all independent variables into the equation. According to the degree of influence of independent variables on dependent variables, the correlation test is used to eliminate the independent variables that are not significant, and the final screened independent variables and dependent variables are established to produce a regression model with correlation [38]. The corresponding formula is as follows:
  $$Y=\beta_{0}+\beta_{1}X_{1}+\beta_{2}X_{2}+\cdots +\beta_{6}X_{6}+\varepsilon$$ (5)
  Among them, Y is the dependent variable, which represents the attention in the new coronavirus network, *X*_1_ represents the number of Internet users in the field of information, *X*_2_ represents the population at the end of the year, *X*_3_ represents the proportion of the population aged 15–64, *X*_4_ represents the proportion of the population aged over 65, *X*_5_ represents the proportion of the population with a high school education, *X*_6_ represents the proportion of the population with a college education and above, *β_i_* is the regression coefficient, and *ε* is the residual and obeys the normal distribution.

### 2.2 Data source

Baidu is the world’s largest Chinese search engine and is committed to providing Internet users with more convenient access to information that they want. Because it is a data sharing platform, we can clearly determine the number of Internet users associated with certain keywords and the development trends of some popular topics over a given period of time. The Baidu index has been applied by many scholars because of its accuracy and convenience of data acquisition [39–41].

Based on the keyword ’novel coronavirus’, the daily average search volume was obtained from January 9, 2020, to April 15, 2020, with the Baidu index platform as the basic dataset. The data collection area included 31 provincial-level administrative regions (excluding Hong Kong, Macao and Taiwan).

The start time of the study was January 9, 2020, because of the outbreak of unexplained pneumonia in Wuhan. The National Health Commission organized multiple units to detect case samples for the first time. On January 8, 2020, the new coronavirus was preliminarily confirmed as the pathogen of the epidemic, and the epidemic dynamics were updated daily. The reasoning behind the deadline selection of April 14, 2020, was that the ’Huoshenshan’ and ’Leishenshan’ hospitals, which played important roles during the development of the epidemic, were officially closed on April 15, 2020, indicating that the epidemic in Wuhan had achieved a phased victory.

## 3. Temporal and spatial characteristics of the novel coronavirus attention network

### 3.1 Time differences in the novel coronavirus attention network

**Fig 1:**
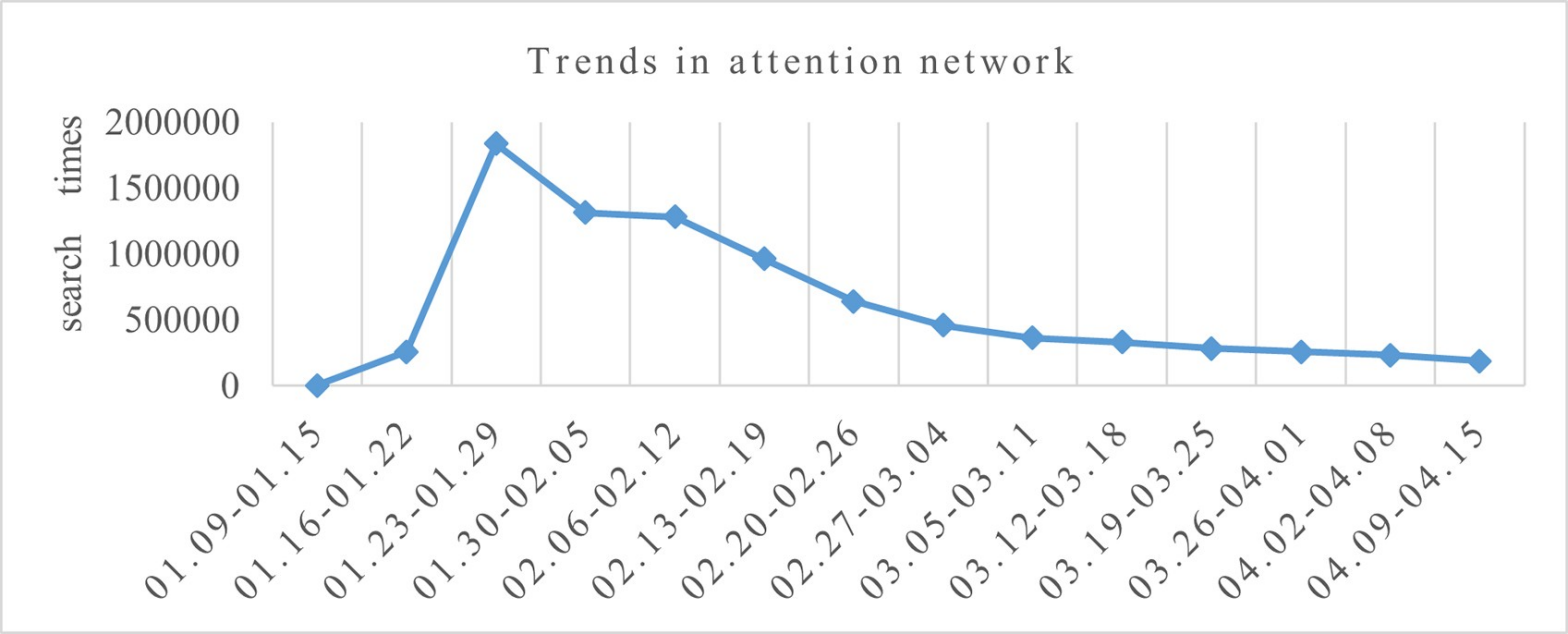
Trend of new coronavirus network attention in China.

A statistical analysis of the novel coronavirus attention network from January 9, 2020-April 15, 2020, showed that the novel coronavirus attention network index exhibited obvious fluctuations during the study period, as shown in Fig 1. From January 9, 2020, to April 15, 2020, the novel coronavirus attention network displayed a sharp growth trend, and the number of searches rapidly increased from 3674 to 1838957. The first case of unexplained pneumonia occurred in Wuhan on December 8th, and the pathogen was identified as a novel coronavirus on January 9, 2020, by an expert group.

On January 20, Dr. Zhong Nanshan confirmed that the new coronavirus epidemic had the phenomenon of ’human transmission’ and that there had been a medical staff member infection. After the closure of Wuhan on January 23, the first-level response to major public health emergencies in Hubei Province was launched on the eve of January 24. Afterward, the ’Huoshenshan’ and ’Leishenshan’ hospitals opened, and medical and nursing workers were sent from across the country to assist in Wuhan. Within a week, with the introduction of various national anti-epidemic measures and publicity from media such as news media, the public’s attention in the new coronavirus network increased explosively. On January 29, the public’s attention in the new coronavirus network reached its peak. The reasons for the peak include that January 29 was the last day of the Spring Festival holiday, and people paid more attention to the characteristics and dissemination of the epidemic after the holiday. Additionally, because of the series of measures enacted previously, people had a basic understanding of the situation of the epidemic.

One week after the peak on January 29, the attention to the new coronavirus continued to decline. The decline in this stage was because there was little change in people’s attention in the new coronavirus network after the peak. By February 5, it dropped to the attention level before the closure of Wuhan, and the daily search volume remained at approximately 1.2 million times. This attention level continued until February 15. The above change trend was because with the development of the epidemic, the response measures were steadily advancing, and there were no incidents that led to a sharp increase in the public’s attention to the epidemic. However, because the epidemic was far from over, the public’s attention to the epidemic had been relatively stable, indicating that the development of the epidemic was relatively stable in this period of time and did not cause great panic among the people. At the same time, the public did not slacken the prevention and control of the epidemic.

With the help of the ’Huoshenshan’ and ’Leishenshan’ hospitals, Wuhan International Convention and Exhibition Center was transformed into a ’field hospital’, and China achieved remarkable anti-epidemic results. Then, on March 11th, President Xi Jinping visited Hubei Province to investigate the prevention and control of the new coronavirus pneumonia epidemic. On March 25, the control of the roadways from Hubei was gradually relaxed, except for roads to and from Wuhan. Due to the timely adoption of appropriate epidemic prevention measures in China, the epidemic prevention and control effects were positive. After January 30, the novel coronavirus attention network displayed a downward trend. By March 25, the novel coronavirus attention network returned to the form observed before the Wuhan lockdown. From March 25 to April 15, the public’s attention to the novel coronavirus began to subside, suggesting that through the efforts of Chinese citizens and the united response to the epidemic, the epidemic situation in China was continually improving. Notably, orderly work and life resumed, and the attention given to the epidemic remained at a stable level (see Fig 1).

The standard deviation and CV of the novel coronavirus attention network from January 9, 2020, to April 15, 2020, are shown in Table 1. During the 14-week period, the difference in network attention was significant. The CV in the second week (January 16–January 22) was the largest, followed by that in the first week. Although the CV fluctuated each week after the third week, it remained stable, and there was no significant change. This result suggests that the trends during the previous two weeks were due to changes in public awareness and anti-epidemic situations or the effects of news, which resulted in novel coronavirus attention network differences. After the third week, on January 23, the city of Wuhan was closed, the epidemic situation intensified, anti-epidemic policy was introduced, and the epidemic became universal. The public began to pay attention to the epidemic progress each day, and the time differences in the attention network gradually narrowed.

**Table 1:** 2020 novel coronavirus network variations from January 9 to April 15.

Week	Average	Standard deviation	Coefficient of variation
**Week 1**	3674.285	2420.089	0.659
**Week 2**	261260.714	328730.976	1.258
**Week 3**	1838957.000	369430.321	0.201
**Week 4**	1314576.429	65877.755	0.050
**Week 5**	1283733.571	264533.942	0.206
**Week 6**	963495.714	152186.698	0.158
**Week 7**	643401.857	105280.877	0.164
**Week 8**	460105.428	33787.737	0.073
**Week 9**	365938.857	24541.372	0.067
**Week 10**	331937.000	24925.557	0.075
**Week 11**	283686.857	12880.166	0.045
**Week 12**	259822.857	12770.540	0.049
**Week 13**	234226.285	37979.949	0.162
**Week 14**	188940.285	4187.883	0.022

### 3.2 Spatial differences in the novel coronavirus attention network

#### 3.2.1 Regional differences in the novel coronavirus attention network

**Fig 2:**
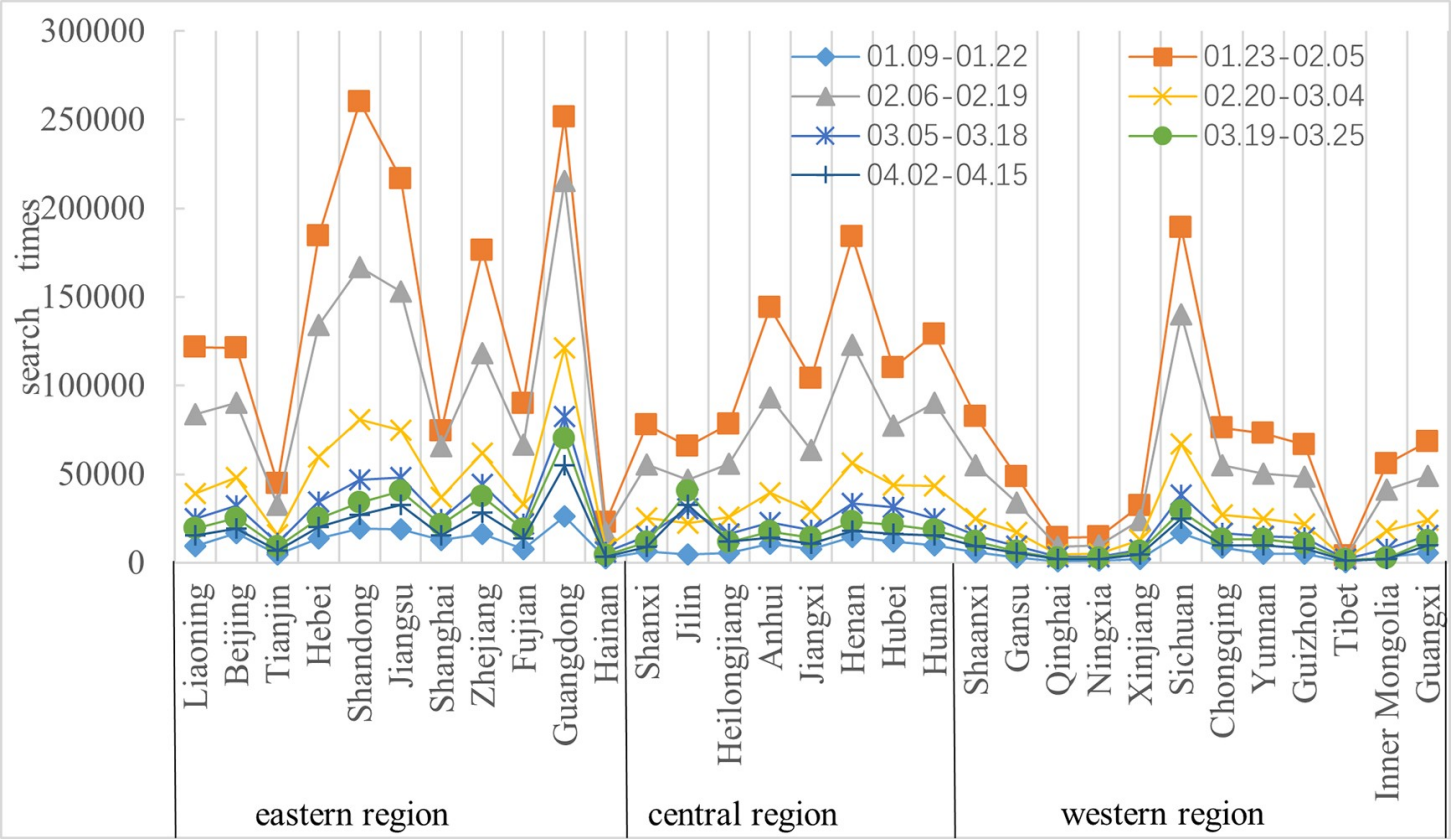
Change trend chart of network attention in 31 provinces.

From January 9, 2020, to April 15, 2020, there were significant differences in the novel coronavirus attention network in the 31 provinces. The attention levels in the main regions peaked in the third week of the epidemic (January 23-January 29) and displayed a downward trend afterward (shown in Fig 2).

According to the interpretation of the National Development and Reform Commission, China’s eastern, central and western division is a policy division in which Inner Mongolia and Guangxi enjoy preferential policies for western development formulated in 2000; thus, Inner Mongolia and Guangxi belong to the western region. From the perspective of the novel coronavirus attention networks in the three regions, the network attention in the eastern and central regions decreased over time, and the network users in the eastern region, especially in Guangdong, Shandong, Jiangsu and other provinces, exhibited the highest attention level toward the novel coronavirus. The attention levels in Tibet, Xinjiang, Qinghai and Ningxia in the western region were among the lowest in China.

The novel coronavirus attention networks from January 9, 2020, to April 5, 2020, in 31 provinces were sorted, and the attention levels were ranked from high to low (see Table 2) based on three classes: 1–10 (high), 11–20 (moderate), and 21–31 (low). Correspondingly, the changes among provinces were divided into stable, fluctuating rise, and fluctuating decline classes.

**Table 2:** Correlation analysis of the factors that influence COVID-19 network attention.

Influential factors			Network attention	
			Correlation	Significance
**Economic level**	Per capita GDP		0.344	0.066
**Population**	Year-end population		0.918**	0.000
**Network level**	Netizens in the information field		0.941**	0.000
	Internet penetration rate		0.340	0.061
**Social population statistical characteristics**	Standard of culture	Primary school	0.807**	0.000
		Junior high	0.886**	0.000
		Senior high	0.924**	0.000
		College or higher	0.939**	0.000
	Age structure	0–14 years	0.809**	0.000
		15–64 years	0.932**	0.000
		65 years and older	0.879**	0.000

Note: ** represent significance at levels 5%.

**Fig 3:**
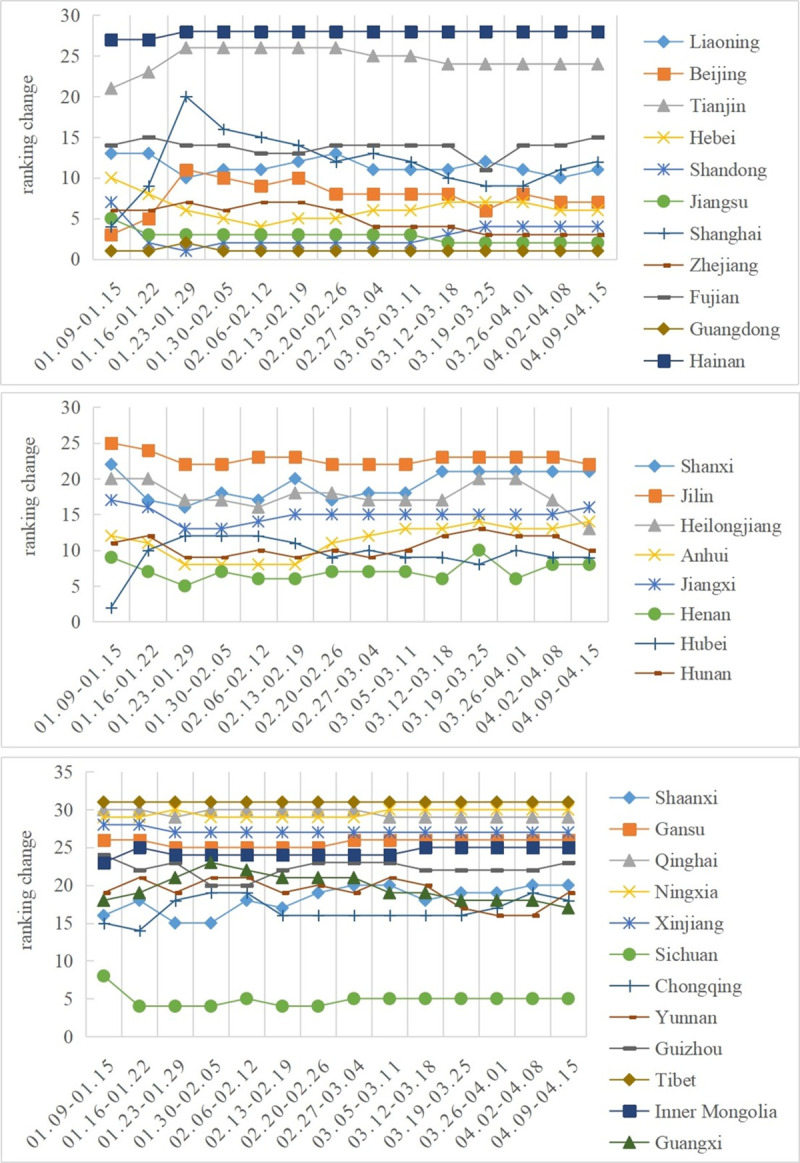
Sequence changes in the network attention level in 31 provinces. a: Sequence change in network attention in the eastern region. b: Sequence change in network attention in central region. c: Sequence change in network attention in the western region.

According to Fig 3, most of the eastern and western regions belong to the stable type, and only four provinces in the central region (Jilin, Jiangxi, Henan, Heilongjiang) belong to the stable type. There are three provinces in the central region (Shanxi, Anhui and Hunan) and only two provinces in the eastern and western regions, namely, Liaoning, Hebei, Yunnan and Guizhou. Beijing and Shanghai in the eastern region, Hubei in the central region and Guangxi in the western region belonged to the type of fluctuation decline. According to the above analysis, we can see that most of the provinces were the stable type in the study period, and the rank changes in several provinces with rising and falling fluctuations are not particularly large, indicating that people’s attention in the new coronavirus network was at a normal level and there were no large fluctuations. This also indirectly shows that China’s various measures in the process of dealing with the epidemic were carried out in an orderly manner and that there was no great panic among the people.

#### 3.2.2 Interprovincial differences in the novel coronavirus attention network

**Fig 4:**
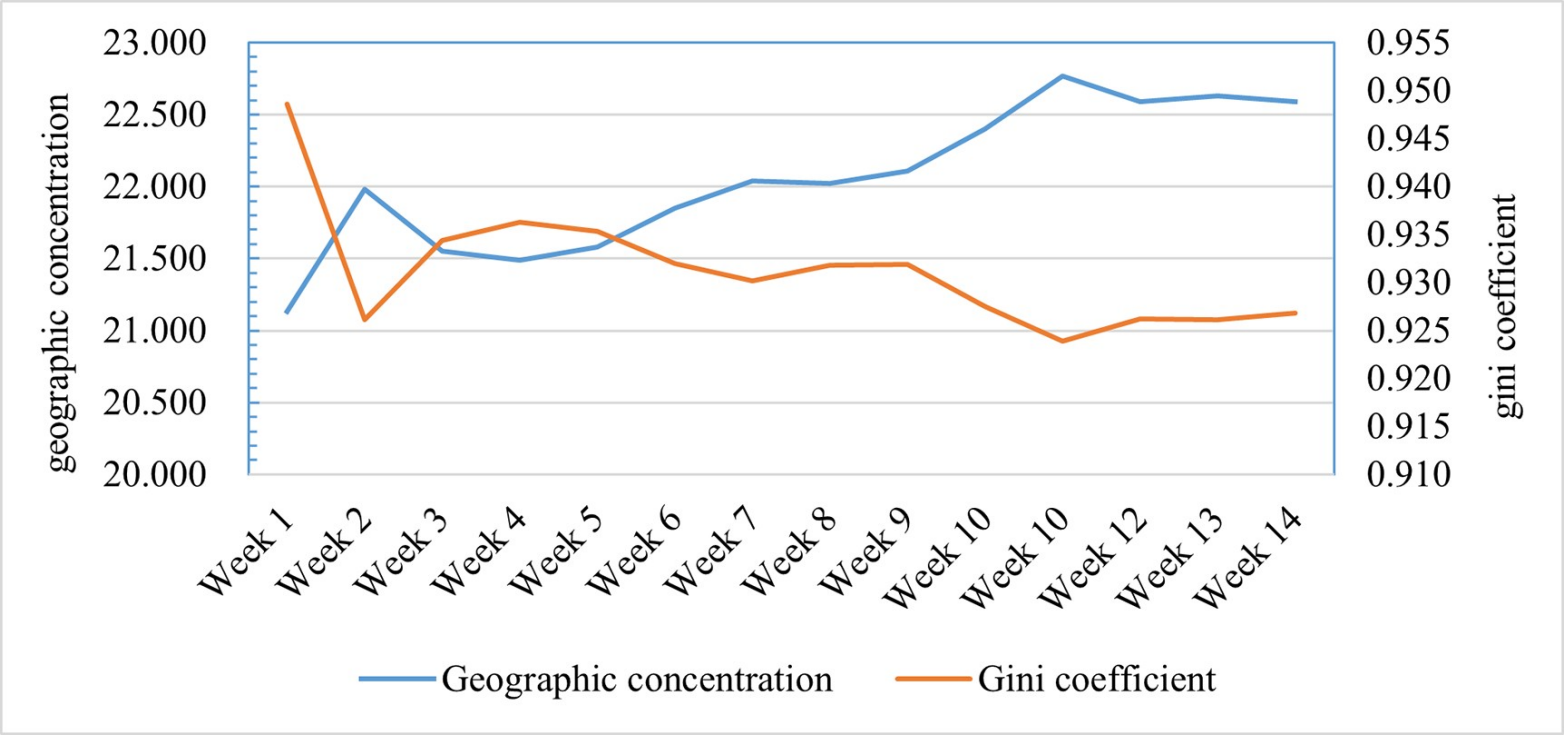
Novel coronavirus network geographic concentration index and Gini coefficient.

To further analyse the interprovincial differences in the novel coronavirus attention network, the geographic concentration index (Ge) and Gini coefficient (Gi) were calculated (see Fig 4). The geographic concentration index encompasses attention fluctuations from January 9, 2020, to April 15, 2020. The changes in this index were small, and it remained basically stable, with values ranging from 21–23, which are far less than 100. In general, it can be concluded that the spatial distribution of the novel coronavirus attention network was not concentrated and was generally decentralized. However, the Gini coefficient did not notably vary but was greater than 0.92, which reflects the local spatial distribution of the novel coronavirus attention network. Based on the above analysis, it can be concluded that the novel coronavirus attention network exhibited ’overall dispersion and local agglomeration’ characteristics. ’Overall dispersion’ was analysed from the national perspective, and the novel coronavirus attention network was divided into 31 provinces. ’Local agglomeration’ included high agglomeration in Shandong, Anhui, and Jiangsu and low agglomeration in Xinjiang, Gansu, and Qinghai.

**Fig 5:**
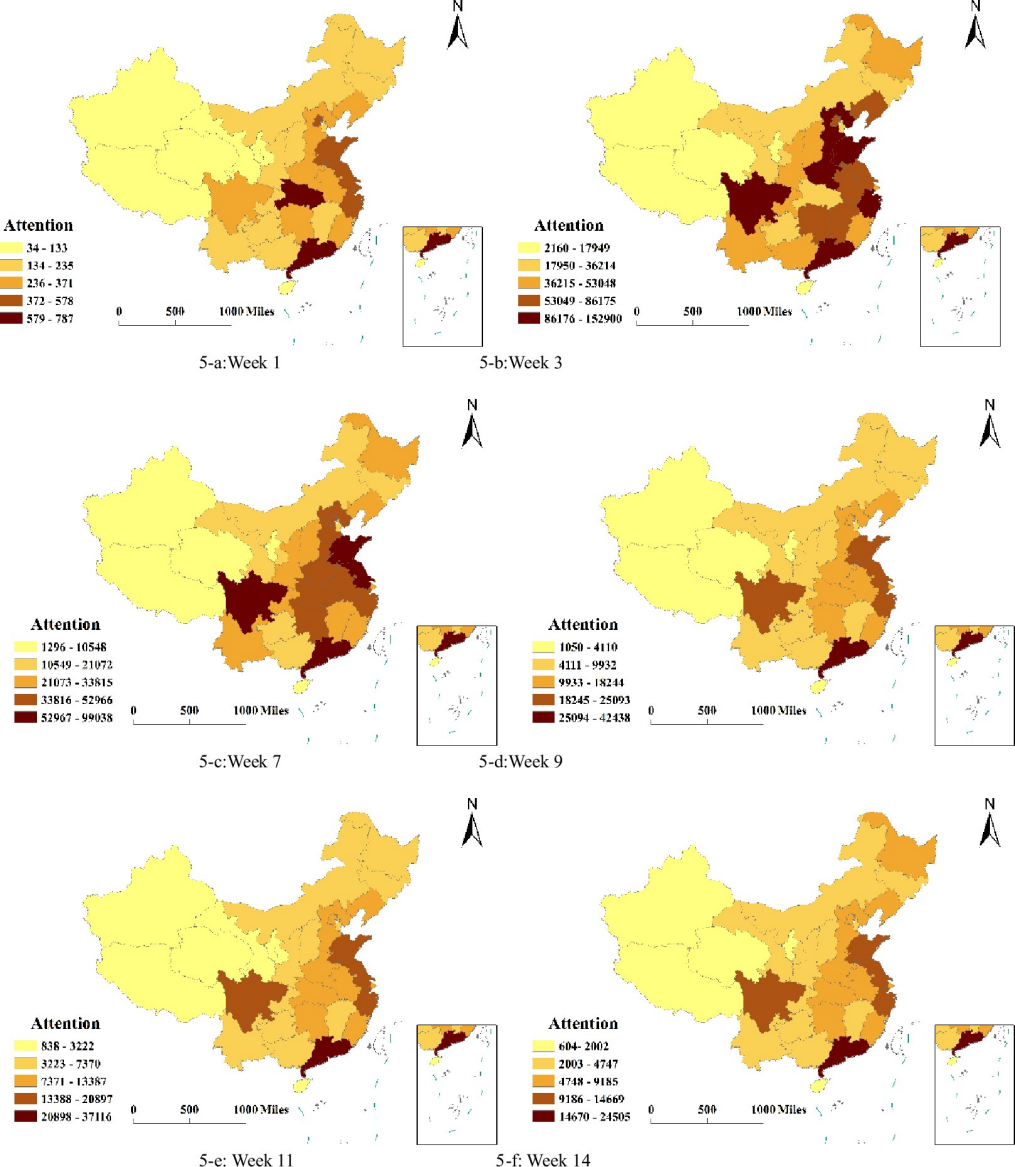
Spatial distribution characteristics of network attention. Source: The primitive base map from the website of the Ministry of Natural Resources of China (http://bzdt.ch.mnr.gov.cn/) was plotted by ArcGIS 10.2 software (ESRI®), and its Drawing Review Number is GS (2016) 2893.

ArcGIS10.2 software was used to draw the provincial spatial distribution map of the network attention of the new coronavirus (Fig 5). Six different time periods were selected to analyse the differences in network attention. These six time periods were the first week, third week, seventh week, ninth week, eleventh week and fourteenth week. The network attention is divided into 1–5 grades according to the depth of colour from low to high. The deeper the colour of the province is, the higher the network attention. The reason for selecting these six periods was that the first week was the initial stage of the epidemic, and the third week to the seventh week were the most serious period during the development of the epidemic. After the ninth week, the epidemic situation was basically stable, and people’s attention to the epidemic was at a relatively stable state afterward.

The following conclusions can be drawn from the figure. First, Hubei and Guangdong were the provinces with the deepest colour in the first week, and the provinces with the deepest colour became more numerous in the third and seventh weeks. In the ninth week, only Guangdong belonged to the type with the deepest colour. This change shows that since the epidemic first occurred in Wuhan City of Hubei Province, Hubei Province was at the fifth level of network attention in the first week. Guangdong Province was at the fifth level of network attention because of its large population, developed economy and rapid information dissemination. With the aggravation of the epidemic situation, in the third week and the seventh week, the number of provinces at the fifth level increased significantly. However, after the ninth week, with China’s positive and effective response measures, the epidemic situation was basically stable, and Guangdong was the only province with the fifth level of network attention.

Second, it can be seen from the graph that most of the eastern provinces and central provinces were at the fourth and third levels of network attention, and this level included only Sichuan Province among the western provinces. The reason for this phenomenon is that these provinces are relatively developed, and the speed of information dissemination is fast. According to the analysis of Fig 5, the level of new coronavirus network attention was highly consistent with the Hu Huanyong line, which is China’s population boundary. The area east of Hu Huanyong line is densely populated, and the network attention was high, mostly in the third to fifth grades. The provinces west of the Hu Huanyong line had low levels of network attention, mostly in the first and second grades.

There was also a phenomenon in Hubei Province. This province experienced the first outbreak of the new coronavirus, and its epidemic situation was the most serious. In the first week, attention in Hubei Province was at the fifth level, which is the highest degree of network attention. Beginning in the third week, in Hubei Province, the level of network attention began to decline compared with the first week. This phenomenon did not indicate that people’s attention in the new coronavirus network decreased in Hubei Province, instead indicating that with the aggravation of the epidemic situation, nationally, people’s attention to the epidemic became generally strengthened, which shows that in the process of epidemic prevention and control, with the participation of the whole population, the collective consciousness is enhanced.

### 3.3 Spatial correlations and clustering in the novel coronavirus attention network

Moran’s I index was used to test the spatial correlations and agglomeration in the novel coronavirus attention network. The research period was half of a month after the lockdown of Wuhan on January 23, and the novel coronavirus attention network of 31 provinces in China was considered. This period was selected because of the explosive growth of the epidemic in the half-month from January 23 to February, and the public’s attention to the epidemic also peaked during this period; therefore, the network attention level in this period was representative. First, the global Moran’s I index was used to test the spatial correlations in the novel coronavirus attention network with GeoDa software, and the global Moran’s I value was 0.146, with a P value of 0.067. These results indicate that the novel coronavirus attention network displayed few positive spatial correlations, mainly because the spatial differences in network attention among different provinces were large, and the agglomeration degree was not high.

**Fig 6:**
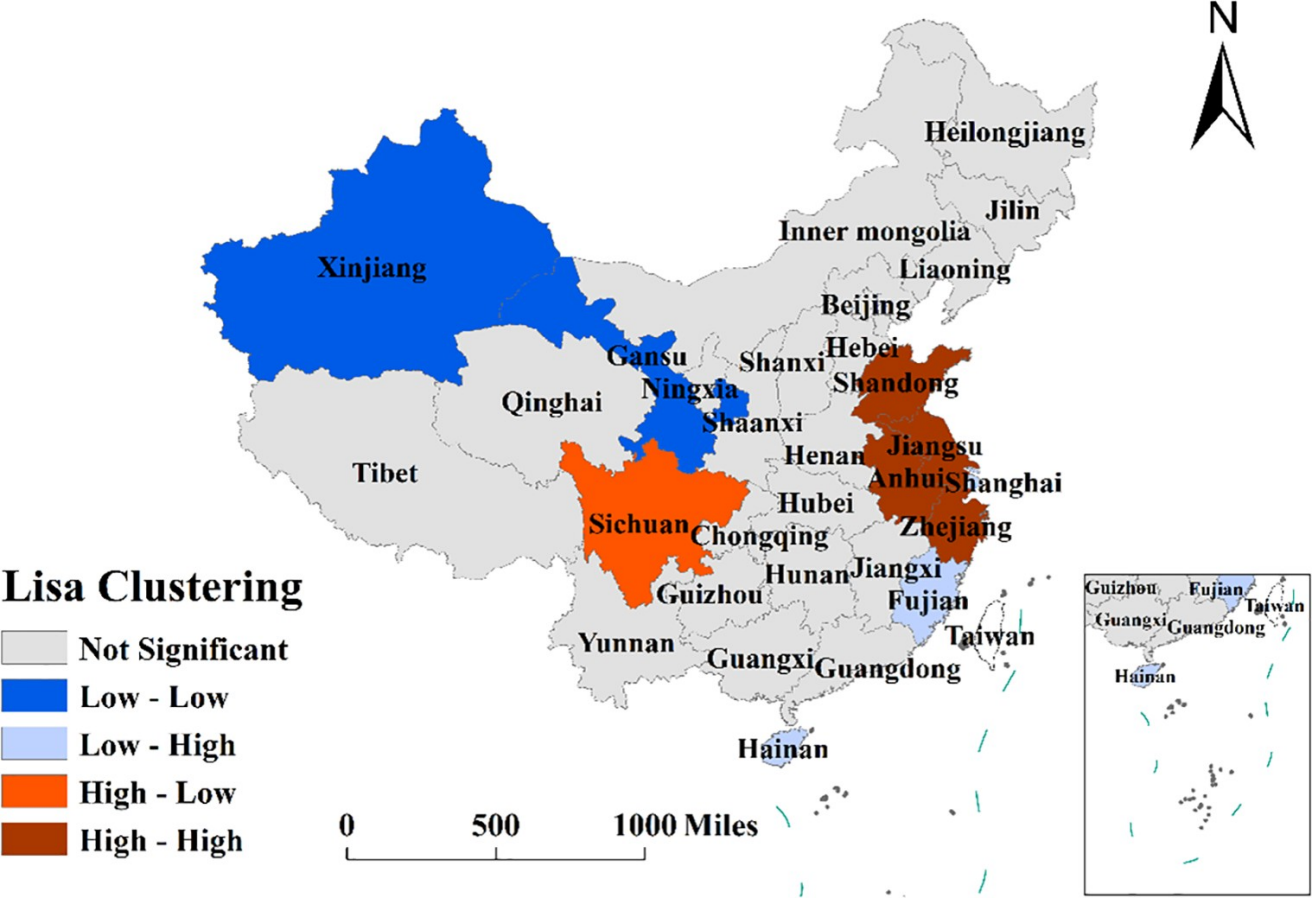
LISA concentration of novel coronavirus network concern. Source: The primitive base map from the website of the Ministry of Natural Resources of China (http://bzdt.ch.mnr.gov.cn/) was plotted by ArcGIS 10.2 software (ESRI®), and its Drawing Review Number is GS (2016) 2893.

Through LISA agglomeration analysis, the spatial agglomeration characteristics of the attention level of the novel coronavirus network were obtained (see Fig 6). It is obvious that these characteristics were not significant, although the agglomeration characteristics of some provinces in the eastern region are obvious. High-high concentration areas (H-H) of the novel coronavirus network were mainly distributed in Shandong, Jiangsu, Anhui and Jiangxi Provinces. The regions with low-low network attention (L-L) were mainly distributed in Xinjiang and Gansu. Sichuan Province was located in the ’V’ type cluster, and it displayed a high network attention level, although the levels of the surrounding provinces were low. The LH-type agglomeration area, namely, the ’A’ type agglomeration area, was mainly distributed in Hainan, Fujian and Shanghai. In contrast to the trend in Sichuan Province, these areas gave limited attention to the novel coronavirus, while the surrounding provinces had high network attention levels. Combined with the analysis results of Moran’s I index, it can be seen that the attention in China’s new coronavirus network presented ’overall dispersion and local agglomeration’ in terms of spatial correlations. Local agglomeration is mainly reflected in the high-high agglomeration areas in the figure, including Shandong, Jiangsu, Anhui and Jiangxi. The regions with low-low agglomeration in the figure are Gansu and Xinjiang.

## 4. Analysis of the factors that influence the novel coronavirus attention network

Based on the analysis of the spatial and temporal distribution of attention of the new coronavirus network in this paper and combined with relevant research results, the factors affecting the spatial and temporal distribution of attention of the new coronavirus network were analysed. According to the availability of data, the following influencing factors are proposed: the level of economic development, the degree of network development, the population at the end of the year, and the characteristics of social population statistics.

The bases of index selection were as follows. The level of economic development represents the quality of life of residents in a region and their level of purchasing power. The higher the economic level, the better the urban infrastructure and services are, which affect people’s network searches. The regional population is the basic factor included to analyse the level of public network attention in a region. The higher the population, the faster information dissemination is, and the more network names there are. The degree of network development is reflected in the level of informatization in the next region in the era of highly developed modern communication network technology. The network penetration rate can directly reflect the development of the Internet in a region, and the proportion of Internet users in the information field is an important indicator that reflects the network behaviour of network users in the region. The characteristics of the social population are important factors affecting the degree of network attention. From the perspective of the educational level, the higher the education level, the higher the network utilization rate of the people is, allowing them to more effectively grasp the information of hot topics and new ideas. With a higher understanding and dependence on the network, these people more reasonably used the network platform to obtain the latest information on the epidemic. Second, from the perspective of age composition, the 15–64 age group has the largest population, and the population of this age group has the highest utilization rate of the network, constituting the main group of Internet users [42–45].

Based on these factors, this study used the Pearson correlation coefficient to test the correlations between the above four influencing factors and the attention in the new coronavirus network (Table 2).

Furthermore, SPSS software was used to perform scatter plot analysis, and there was a linear relationship between the selected influential factors and the network attention level. A regression coefficient table was obtained through regression analysis (see Table 3).

**Table 3:** Regression coefficients between novel coronavirus attention and influential factors.

Model	Non-normalized beta coefficients	Standardized beta coefficients	T	Sig.	Collinearity statistics	
					Tolerance	VIF
**Message**	6061.970	0.941	14.962	0.000	0.325	3.076
**Population**	4.313	0.918	12.446	0.000	0.352	2.844
**15–64 years**	0.768	0.932	13.842	0.000	0.325	3.076
**aged over 65**	3.812	0.879	9.912	0.000	0.360	2.776
**Senior high**	2.897	0.924	12.981	0.000	0.317	3.151
**College or higher**	4.688	0.939	14.732	0.000	0.119	8.377

### (1) Economic development level

Table 2 shows that although there was a positive correlation between the per capita GDP and network attention, the relation did not pass the significance test. Therefore, economic factors had no significant impact on the attention network in different regions. From the outbreak of the epidemic to the Wuhan lockdown on January 23, various news items were released about the novel coronavirus. In addition, each region started to respond to major public health emergencies, making people realize the urgency and danger of the epidemic. Consequently, the novel coronavirus became a national focus within a very short period of time. Economic factors had a limited influence on the outbreak of new coronavirus pneumonia because the particularity of this outbreak is that it is related to people’s health and safety, and everyone has the responsibility and obligation to understand the development of the epidemic.

### (2) The degree of network development

The degree of network development is one of the indispensable factors related to network attention. This paper studies the degree of network development from two aspects: the proportion of Internet users in the field of information and the Internet penetration rate. From Table 2, it is concluded that the positive distribution of the Internet penetration rate in the novel coronavirus attention network was not obvious, and the proportion of Internet users in the field of information was significantly positively correlated with network attention (r = 0.941, P < 0.05).

These results show that the popularity rate of the Internet in a region could not significantly indicate the level of network attention, and the network behaviour of Internet users in the information field could better reflect people’s search needs. Provinces with a more accurate population and a larger proportion of Internet users in the information field exhibited higher network attention to the new coronavirus. During the epidemic period, the state and the government updated the relevant information and provided the latest progress of the epidemic in a timely manner. The release of information was more accurate and transparent, which was helpful in allowing Internet users to obtain the latest epidemic information.

### (3) Regional population statistics

Since the epidemic of neo-coronal pneumonia is a national public health event and the infectiousness of neo-coronal pneumonia is very strong, the larger the population size of a region is, the greater the risk of epidemic transmission and the more attention people generally pay to events such as epidemics. Table 2 shows that the novel coronavirus attention network was significantly positively correlated with the population at the end of the year (r = 0.918, P < 0.05). The larger the population at the end of the year in a province was, the higher the network attention level. The research data also suggest that Guangdong and Shandong, which were the two provinces with the highest populations at the end of the year, exhibited the highest network attention levels. The more densely populated areas are, the higher the risk of cross-infection is. Therefore, for areas with large populations and high population densities, the risks and challenges in epidemic prevention are also great.

### (4) Social demographic characteristics

This paper analyses the impact of sociodemographic characteristics on network attention from two perspectives: education level and age structure. This paper divides the educational level of the population into four grades: primary school and below; junior high school; high school; and college and above. Table 2 shows that the four levels of education were significantly correlated with network attention, and the higher the education level was, the more significant the correlation was. The significance of junior college and above education (r = 0.939, P < 0.05) was the highest, exhibiting a strong correlation and indicating that the level of education was one of the important factors that influenced the novel coronavirus attention network. The higher the education level, the higher the utilization rate of the Internet, and the higher the dependence on the network. To assess the potential impacts of age, this paper divides the age structure of the population into three levels: 0–14 years old, 15–64 years, and over 65 years old.

From the analysis results, it was concluded that the population distribution in the three age groups was significantly correlated with network attention. The correlation coefficient (r = 0.932, P < 0.05) between the 15-64-year-old age group and network attention was the largest, and the correlation was strong. The reason for this phenomenon is that the population base of this age group is relatively large, thus leading to a high network attention level. The population of this age group is the main group of Internet users. Additionally, the age group over 65 years old (r = 0.879, P < 0.05) was highly threatened by the novel coronavirus. The novel coronavirus is a new virus that was discovered in 2019 and has never previously been found in the human body. There were no antibodies in the population, so all humans were susceptible. The most susceptible population is the elderly population, especially individuals with low immunity and chronic basic diseases. Thus, many people over 65 years old have paid special attention to novel coronavirus news.

## 5. Discussion

The global pandemic of COVID-19 has asserted the widest impact on humans in the past 100 years and is a serious crisis and severe test worldwide. Human safety and health are facing major threats. As of June 21, 2021, 31 provinces reported 117548 confirmed cases, 99247 cured cases and 5395 deaths. In total, 179520170 patients have been diagnosed according to foreign epidemic data. In total, 150027808 patients have been cured, and 3879040 patients have died.

China has paid great costs and sacrifices. The people of the whole country are united and initially curbed the spread of the epidemic in more than one month. It took approximately two months to control the daily new cases in local areas to fluctuations within a single digit. It took approximately three months to achieve decisive results in the Wuhan and Hubei control strategies. Prevention and control of the epidemic in China has achieved major strategic results, maintained the safety of people’s lives and health, and made important contributions to the maintenance of regional and worldwide public health security. Based on theoretical studies of the spatial-temporal distribution and influencing factors of the new coronavirus network attention and the series of important measures taken during epidemic prevention in China, this paper puts forward the following suggestions and experiences [46].

1. To establish a unified and efficient command system, in the process of epidemic prevention and control, China can quickly respond to sudden outbreaks, including the timely introduction of countermeasures, mobilization all forces, and exhibition of unity [47]. Through the popular science professional platform, the media and the Internet, government departments popularize scientific cognition, distribute scientific prevention and control knowledge to the public, organize authoritative experts to introduce daily prevention and control knowledge, guide the public to rationally understand the epidemic of new coronavirus pneumonia, and perform well in personal protection and elimination of panic and fear [48].
2. First, we should use big data and artificial intelligence to judge the development trend of the epidemic. For example, in this paper, the Baidu index is used to reflect the level of people’s attention in the new coronavirus network in different regions and different periods and to scientifically analyse the future development of the epidemic in different regions, which is of great help and reference significance for the government in implementing precise prevention and control policies. Second, full play should be given to the supporting role of science and technology by using scientific and technological forces to carry out scientific prevention and control and accelerate the development and application of drugs, vaccines, detection reagents, etc.. Populations with underlying conditions in countries and regions should be vaccinated as soon as possible and adhere to the combination of scientific research, clinical treatment, and prevention and control practices [49].

## 6. Conclusion

After the outbreak of the novel coronavirus epidemic, China immediately took the appropriate response measures to effectively protect people’s lives. As individuals involved in epidemic prevention and control, we should actively pay attention to the most recent developments regarding the epidemic and abide by the principles of epidemic prevention and control. Taking the new coronavirus network attention as the research object, this paper discusses the spatial and temporal differentiation and influencing factors of new coronavirus network attention by means of geographic concentration, Gini coefficient, spatial autocorrelation and multiple stepwise linear regression. The main conclusions are as follows.

1. Temporally, the attention in the new coronavirus network showed an upward trend from January 9 to January 29, with the largest increase from January 23 to January 29 and a peak on January 29, after which it showed a slow downward trend. The level of attention in the new coronavirus network was basically flat when comparing March 4 and January 22, and the level of attention remained stable level afterward.
2. From a regional perspective, China’s new coronavirus network attention showed a decreasing trend from east to west. The highest attention in the east was in Shandong Province and Guangdong Province, the highest attention in the middle area was in Henan Province, and the highest attention in the west was in Sichuan Province. From the overall distribution of network attention, the level of regional network attention in the east of the Hu Huanyong line was generally high, while the level of regional network attention in the west of the Hu Huanyong line was low.
3. From the perspective of the spatial correlations and clustering of new coronavirus network attention, the high-high clustering area (HH) of new coronavirus network attention was mainly distributed in Shandong, Jiangsu, Anhui and Jiangxi provinces, which are hot provinces. The regions with low-low network attention (LL) were mainly distributed in Xinjiang and Gansu, which are cold areas. Sichuan Province exhibited high attention in the new coronavirus network, while the surrounding provinces exhibited low attention in the new coronavirus network. In contrast to Sichuan Province, Fujian and Shanghai showed low attention in the new coronavirus network, while their surrounding provinces showed high attention in the new coronavirus network.
4. Through the analysis of influencing factors, it was concluded that the population at the end of the year, the proportion of Internet users in the information field, and the educational level and age structure of the population were the factors affecting the attention in the new coronavirus network. Population was the most basic factor that indicated the level of network attention. The larger the population of a province was, the higher the network attention was. The number of Internet users in the information field could more accurately reflect the network behaviour of regional Internet users. The population aged 15–64constitutes the main group of network users in cities. The higher the education level of the population is, the stronger the dependence on and use of the network will be. Therefore, these two characteristics of the social population were also important factors affecting the attention in the new coronavirus network.

In this paper, the public’s attention level in the new coronavirus network in different regions was obtained through Baidu big data. Through the study of network attention, the reflection and attention of each region to the epidemic could be judged. The characteristics of the new coronavirus network attention were analysed from the perspective of time and space, and the influencing factors were also analysed. The findings can provide scientific guidance for the normalization of the prevention and control of epidemics and other major public health emergencies in the future, and prevention and control policies can be made timelier and more accurate.

This article has some shortcomings. For example, the time span of the article was small, and analyses can be refined from the provincial level to the city and county levels. Additionally, the data collection channels were relatively limited, and using the Internet, people’s attention levels can be monitored through different channels, such as Sina Weibo, short video browsing, and Google searches. Moreover, subdata analysis can reflect people’s attention levels. The selection of the keyword was relatively simple in this study, and only ’novel coronavirus’ was used. During the epidemic, search terms such as case data and novel corona symptoms were also keywords of interest. Finally, the selection of influential factors in the novel coronavirus attention network was based on the availability of data, and the selection range was narrow; therefore, the selection of these factors and indicators needs to be studied further.

## Supporting information

S1 Data (RAR)
